# Unraveling of Functional Activity of Primary Hippocampal Neuron-Glial Networks in Photodynamic Therapy Based on Tetracyanotetra(aryl)porphyrazines

**DOI:** 10.3390/cells11071212

**Published:** 2022-04-04

**Authors:** Maria O. Savyuk, Victoria D. Turubanova, Tatiana A. Mishchenko, Svetlana A. Lermontova, Larisa G. Klapshina, Dmitri V. Krysko, Maria V. Vedunova

**Affiliations:** 1 Department of Basic and Medical Genetics, Institute of Biology and Biomedicine, Lobachevsky State University of Nizhny Novgorod, 23 Gagarin ave., 603022 Nizhny Novgorod, Russia; mary.savyuk@bk.ru (M.O.S.); vikaturu@mail.ru (V.D.T.); saharnova87@mail.ru (T.A.M.); dmitri.krysko@ugent.be (D.V.K.); 2 Department of Neurotechnology, Institute of Biology and Biomedicine, Lobachevsky State University of Nizhny Novgorod, 23 Gagarin ave., 603022 Nizhny Novgorod, Russia; 3 Sector of Chromophors for Medicine, G.A. Razuvaev Institute of Organometallic Chemistry of the Russian Academy of Sciences, 49 Tropinin st., 603137 Nizhny Novgorod, Russia; lermontovasa@rambler.ru (S.A.L.); klarisa@ioms.ras.ru (L.G.K.); 4 Cell Death Investigation and Therapy Laboratory (CDIT), Department of Human Structure and Repair, Ghent University, C. Heymanslaan 10, Building B3, 4th Floor, 9000 Ghent, Belgium; 5 Cancer Research Institute Ghent, 9000 Ghent, Belgium

**Keywords:** photodynamic therapy, porphyrazines, brain cancer, primary neuronal cultures, functional neural network activity, calcium activity

## Abstract

The current efforts in photodynamic therapy (PDT) of brain cancer are focused on the development of novel photosensitizers with improved photodynamic properties, targeted specific localization, and sensitivity to the irradiation dose, ensuring the effectiveness of PDT with fewer side effects for normal nerve tissue. Here, we characterize the effects of four photosensitizers of the tetracyanotetra(aryl)porphyrazine group (**pz I**–**IV**) on the functional activity of neuron-glial networks in primary hippocampal cultures in their application in normal conditions and under PDT. The data revealed that the application of **pz I**–**IV** leads to a significant decrease in the main parameters of the functional calcium activity of neuron-glial networks and pronounced changes in the network characteristics. The observed negative effects of **pz I**–**IV** were aggravated under PDT. Considering the significant restructuring of the functional architectonics of neuron-glial networks that can lead to severe impairments in synaptic transmission and loss of brain functions, and the feasibility of direct application of PDT based on **pz I**–**IV** in the therapy of brain tumors is highly controversial. Nevertheless, the unique properties of **pz I**–**IV** retain a great prospect of their use in the therapy of tumors of another origin and cellular metabolism.

## 1. Introduction

Glioblastoma multiforme is the most frequent and aggressive type of malignant tumors of the central nervous system with extremely low overall patient survival [1,2,3]. Due to the low effectiveness of current treatment methods of gliomas, there is an urgent need to develop breakthrough therapeutic strategies to achieve maximum tumor destruction and decrease the risk of secondary tumors and metastases. The crucial stage in the development of any therapeutic approach for treatment of brain tumors is the evaluation of the effects of anticancer agents on normal nerve cells. Brain cells, especially neurons, have certain metabolic peculiarities, making them extremely sensitive to a toxic load. The death of neuron-glial network elements or changes in their functional activity can significantly affect brain functions, which can aggravate the patient’s condition and negate the effectiveness of the therapy.

Photodynamic therapy (PDT) includes in the list of perspective tools for the treatment of gliomas. PDT is a multi-step procedure that combines systemic or local administration of a specific agent with photodynamic activity (i.e., photosensitizer), followed by its accumulation in the tumor tissue and excitation by irradiation with a light of appropriate wavelength and subsequent in the presence of cellular oxygen generation of cytotoxic reactive oxygen species [4,5,6,7,8]. In recent years there has been a revival intertest to PDT because it is an attractive and effective anti-cancer approach due to its low invasiveness, low toxic effect for healthy tissues, and the possibility of its use both as an independent therapy and in combination with other treatment modes (e.g., chemotherapy, photothermal therapy) [9]. Moreover, it has been shown that, during PDT, several photosensitizers can induce the immunogenic pathway of cancer cell death, so-called immunogenic cell death (ICD) resulting in the emission of damage-associated molecular patterns (DAMPs), which act as danger signals and adjuvants to activate the anti-tumor immunity. The release of DAMPs facilitates the cross-presentation of antigenic peptides on major histocompatibility complex class I (MHC I) molecules to CD8^+^ T cells of the adaptive immune system and generation a long-lasting immunological memory able to eliminate the tumor cells that survived during initial therapy and, therefore, achieve a complete tumor eradication [7,10,11,12].

Currently, PDT is widely used in clinical practice in the treatment of tumors of various origins, including brain tumors. For instance, PDT based on hypericin [13], 5-ALA [14], hematoporphyrin derivative [15], photofrin [16], and photosens [17] is known to be efficient against gliomas. However, despite PDT being regarded as a useful tool for cancer treatment, several persistent challenges compromise its efficiency. From the viewpoint of the central nervous system, the ability of the photosensitizer to penetrate normal cells preserves the risk of developing toxic effects leading to the destruction of neuron-glial networks and significant changes in their functional architectonics under photodynamic exposure. Therefore, there is still a need for the development of photosensitizers with improved photodynamic properties, targeted specific localization, and sensitivity to the irradiation dose, ensuring the effectiveness of PDT with fewer side effects.

We recently presented a novel group of tetracyanotetra(aryl)porphyrazines (hereinafter, **pz**) as promising photosensitizers for PDT [18,19]. These compounds have unique properties providing an elegant combination of photophysical and cytotoxic properties (e.g., stable production of cytotoxic singlet oxygen), which function as sensors and molecular rotors. The action of these photosensitizers is dependent on fluorescence of the rate of intramolecular rotation that opens up an ability to detect tumor cells’ sensitivity to photoinduction and timely optimizes the treatment modes for each patient, allowing for the opportunity to have more personalized cancer therapy [18]. We have previously shown the effectiveness of four **pz** (**pz I**–**IV**) in PDT treatment of glioma in vitro. It was shown that **pz I**–**IV** accumulated in both healthy neuronal cells and glioma cells, but the rate of their internalization, subcellular localization, and dark toxicity differed significantly [19].

In the present study, we studied the features of the functional activity of neuron-glial networks in primary hippocampal cultures in the application of **pz I**–**IV** with (PDT) and without (in the dark) photodynamic exposure.

## 2. Materials and Methods

### 2.1. Ethics Statement

The animals were housed in a certified SPF vivarium of Lobachevsky State University of Nizhny Novgorod. All experimental procedures were approved by the Bioethics Committee of Lobachevsky University and carried out in accordance with Act 708n (23 082010) of the Russian Federation National Ministry of Public Health, which states the rules of laboratory practice for the care and use of laboratory animals, and the Council Directive 2010/63 EU of the European Parliament (22 September 2010) on the protection of animals used for scientific purposes. Pregnant C57BL/6 mice (day of gestation 18) were sacrificed by cervical vertebra dislocation.

### 2.2. Isolation of Murine Primary Hippocampal Cultures 

Primary hippocampal cultures were obtained from mice embryos and cultured on culture plates pretreated with polyethyleneimine solution (1 mg/mL) (Sigma-Aldrich, Darmstadt, Germany) according to the previously developed protocol [20]. Isolation of embryonic hippocampi was performed in Ca^2+^- and Mg^2+^-free phosphate-buffered saline (PBS, Invitrogen, Waltham, MA, USA) with subsequent enzymatic digestion with 0.25% trypsin-ethylenediaminetetraacetic acid (EDTA, Invitrogen) for 20 min. After centrifugation (800 rpm for 3 min), the cell suspension was seeded on a culture plate at an approximate initial density of 7000–9000 cells/mm^2^. The primary hippocampal cultures were grown in Neurobasal medium (Invitrogen) supplemented with 2% B27 (Invitrogen), 0.5 mM L-glutamine (Invitrogen), and 0.4% fetal bovine serum (FBS; Biosera, Nuaillé, France) under constant conditions of 37 °C, 5% CO_2_, and a humidified atmosphere in a Binder C150 incubator (BINDER GmbH, Tuttlingen, Germany). Half of the medium was replaced once every three days.

According to modern paradigm, neural networks are the minimum functional unit of the central nervous system responsible for the implementation of higher cognitive functions and the ability to respond to environmental changes [21]. Recent experimental evidence suggested that astrocytes capable of forming their own networks and interacting with neurons provide mutual regulation the functional activity of each other [22,23]. Therefore, the realization of brain functions is largely dependent on functional ensembles of neurons and glial cells that form complex neuron-glial networks.

We previously showed that primary hippocampal cultures can serve as a relevant biological model of the brain neuron-glial networks in vitro [24]. In particular, we characterized the cellular content and the features of functional activity of neuron-glial networks in the different period of cultivation in vitro [24,25,26].

The current study was conducted beginning with day 14 of primary hippocampal cultures development in vitro (DIV). This period is characterized by the presence of neurons and glial cells in the cultures in an approximate ratio of 1:2, the prevalence of a population of mature chemical synapses with mature axo-dendritic and axo-spiny asymmetric contacts, and stable functional activity of neuron-glial networks, which has a complex characteristic pattern [24,25,26].

### 2.3. Photosensitizers and Irradiation Modes

**Figure 1:**
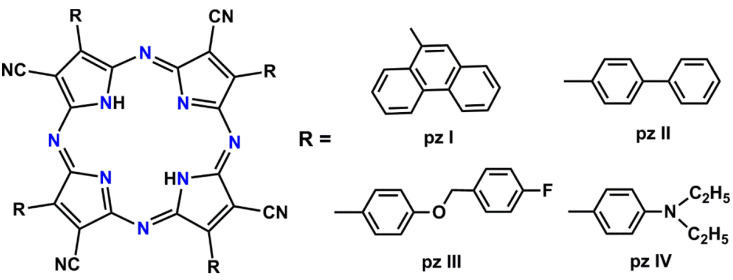
A chemical structure of the synthesized tetracyanotetra(aryl)porphyrazines: **pz I**, **pz II**, **pz III**, and **pz IV**.

Four tetracyanotetra(aryl)porphyrazine dyes with 9-phenanthrenyl (**pz I**), 4-biphenyl (**pz II**), [4-(4-fluorobenzyoxy) phenyl (**pz III**), and 4-diethylaminophenyl (**pz IV**) groups in the periphery of the porphyrazine macrocycle were tested. The photosensitizers were obtained according to the previously described synthetic approach using a metal template assembly of the porphyrazine frameworks [27,28]. The chemical structure of the synthesized porphyrazines is shown in Figure 1.

On day 14 of cultivation (14 DIV), the culture medium was replaced to a serum-free medium containing porphyrazines at concentrations of 1.18 µM for **pz I**, 0.19 µM for **pz II**, and 0.54 µM and 0.37 µM for **pz III** and **pz IV**, respectively. Since we consider **pz I**–**IV** as potential photodynamic agents for PDT treatment of brain tumors, the concentrations we used corresponded to the IC_50_ for murine glioma GL261 cells, which were experimentally established in our previous studies [18,19]. After 4 h of incubation, the medium was replaced with a pz-free complete culture medium. The primary hippocampal cultures were then subjected to photodynamic exposure by light irradiation at the dose of 20 J/cm^2^ using a LED light source (*λ*_ex_ 615–635 nm, 20 mW/cm^2^). For the dark toxicity estimation, the cells loaded with **pz I**–**IV** were stored in the dark for an equal time as PDT groups. The primary hippocampal cultures that did not receive a **pz I**–**IV** addition to the culture medium served as a control.

### 2.4. Functional Calcium Imaging

To expand our knowledge about the pathological processes in the central nervous system or assess the effect of a therapeutic agent, it is essential to study the functional activity of neuron-glial networks. The calcium imaging technique provides registration of the spatiotemporal patterns of neural-glial network activity. Visualization of calcium dynamics in the cytoplasm of nerve cells is an extremely informative approach for assessing neural-glial network metabolic activity since it allows for visualizing the architecture and mapping the activity of networks with cellular resolution [26].

Functional calcium imaging was performed on the next day after photodynamic therapy according to the previously developed protocol [29]. A fluorescent calcium-sensitive dye Oregon Green 488 BAPTA-1 AM (Invitrogen) was dissolved in DMSO with 4% pluronic F-127 (Invitrogen), and then added to the medium of the primary hippocampal cultures followed by 20 min incubation in a CO_2_-incubator. The obtained samples were examined using an LSM 800 confocal laser scanning microscope (Carl Zeiss, Oberkochen, Germany). The OGB1 fluorescence excitation wavelength was set at 488 nm with an argon laser, and the emission was recorded in the range of 500 to 530 nm. 

To evaluate the dynamics of changes in the intracellular calcium concentration, time series of confocal images were recorded. The registration rate used was three frames per second. The following parameters were analyzed: the duration of calcium oscillations (the time period from the beginning to the end of an oscillation, s), the frequency of calcium oscillations (an average number of oscillations per min), and the percentage of active cells (the cells number with at least one recorded oscillation divided by the total cell number, %).

### 2.5. Analysis of Network Characteristics of Primary Hippocampal Cultures

We recently developed algorithm providing the analysis of calcium imaging data by using correlation analysis for construction of the dynamical architecture of neuron-glial networks [20,22,26,30]. This approach allowed us to assess the activity both neurons and astrocytes in the network, to assess the collective and coordinated activity of neuron-glial networks on the functional level, and to evaluate the effects of therapeutic agents [20,26,31]. Used algorithm represents the neuron–glial network as an oriented graph of the nodes, which correspond to individual cells, and the edges connect the corresponding nodes and indicate a significant correlation between pairs of cells (*p* > 0.3). The spread of calcium signals between cells results in detecting time delays in the increase in Ca^2+^ concentration. The following parameters were analyzed: the correlation between cells, the average number of connections per cell, the ratio of the available connections in the culture to the maximum possible number of connections in the culture, and the signal speed propagation. This complex group of parameters allowed us to assess the functional state of the neuron-glial networks in the culture.

### 2.6. Statistical Analysis

Statistical analysis was performed using GraphPad Prism v.9.3.1.471 https://www.graphpad.com (accessed on 2 December 2021) (San Diego, CA, USA). Data were expressed as box and whisker plots with Tukey’s method. We performed Kruskal–Wallis non-parametric ANOVA. Difference was considered to be statistically significant if *p* < 0.05. At least three independent biological replicates were used for all experiments.

## 3. Results

We previously characterized the long-term dark toxicity effects of **pz I**–**IV** on the viability of primary neuronal cultures [19]. It was shown that **pz I**–**IV** accumulated in both healthy neuronal cells and glioma cells, but the rate of their internalization, subcellular localization, and dark toxicity differed significantly [19]. In the current study, we focused on the assessment of the features of metabolic functional activity of neuron-glial networks of primary hippocampal cultures in the application of **pz I**–**IV** in the dark and under PDT.

**Figure 2:**
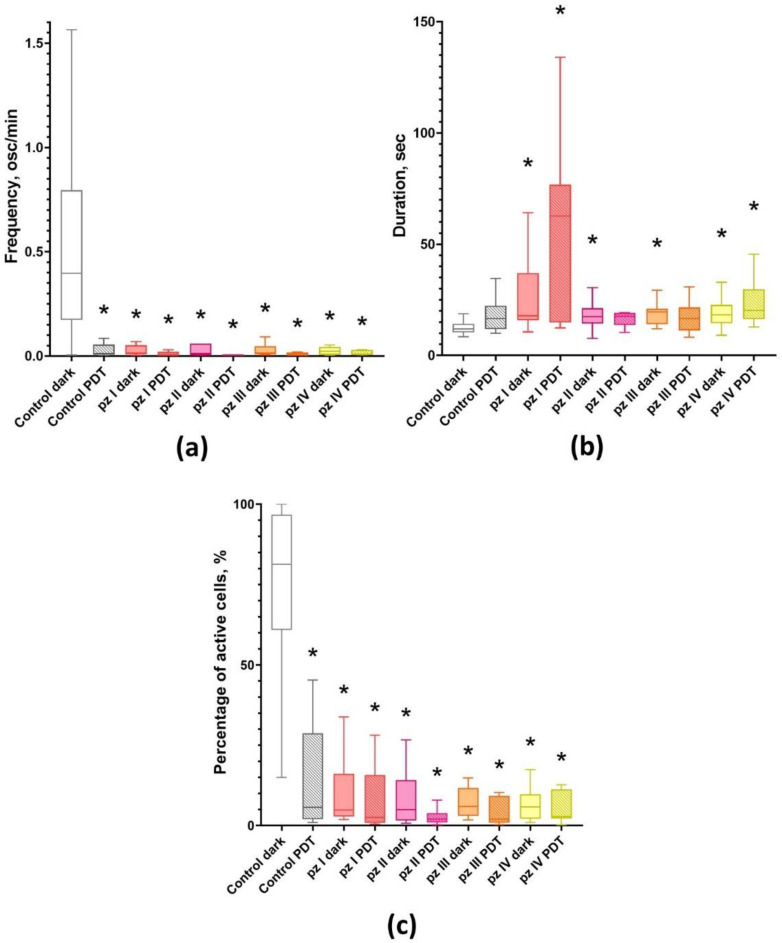
Main parameters of functional calcium activity in primary hippocampal cultures the next day after PDT. (**a**) Number of Ca^2+^ oscillations per min; (**b**) duration of Ca^2+^ oscillations; (**c**) proportion of cells exhibiting Ca^2+^ activity. * versus “Control dark”; *p* < 0.05, Kruskal–Wallis test.

Analysis of the main parameters of calcium activity showed that cultures not exposed to PDT (control dark) have a high percentage of functionally active cells (81.32 [61.28; 95.32] %) (Figure 2). At the same time, the calcium activity profile is characterized by frequent Ca^2+^ oscillations (0.56 [0.21; 0.95] osc/min) of low duration (11.91 [10.61; 13.96] s). As we previously showed, such a profile of the functional calcium activity is typical for this period of cultivation of primary hippocampal cultures in vitro [31].

PDT leads to a significant decrease in the functional calcium activity of neuron-glial networks of primary hippocampal cultures (Figure 2). In the “Control PDT” group, the number of active cells (7.06 [0.38; 20.62] %), as well as the frequency of Ca^2+^ oscillations (0.01 [0.01; 0.04] osc/min) decreased relatively the group “Control dark” by 11.5 and more than 50 times, respectively. 

The application of all studied photosensitizers (**pz I**–**IV**) resulted in inhibition of the functional calcium activity of primary hippocampal cultures either subjected to PDT or not (Figure 2). The observed effect was accompanied by a dramatic decrease in the number of functionally active cells and significant changes in Ca^2+^ oscillations profile including a decrease in the frequency and an increase in the duration of Ca^2+^ oscillations. There were no significant differences between the experimental groups and the “Control PDT” group. Such changes in the Ca^2+^ profile are supposed to be associated with the destruction of synaptic contacts and degradation of axons and dendrites, which in turn can potentially lead to the complete destruction of neural networks and, as a consequence, loss of brain functions.

The no less significant analysis that determines the functional state of primary neuronal cultures is an assessment of the parameters of network activity. In normal conditions, most cells in the neuron-glial network work simultaneously and are connected to each other. The death of functionally important elements in response to a stress factor can lead to the substantial restructuring of the network resulting in its simplification and pronounced functional impairment. The potential death of the neuron-glial network is evidenced by the lack of correlation in the profile of Ca^2+^ oscillations between functionally active cells.

**Figure 3:**
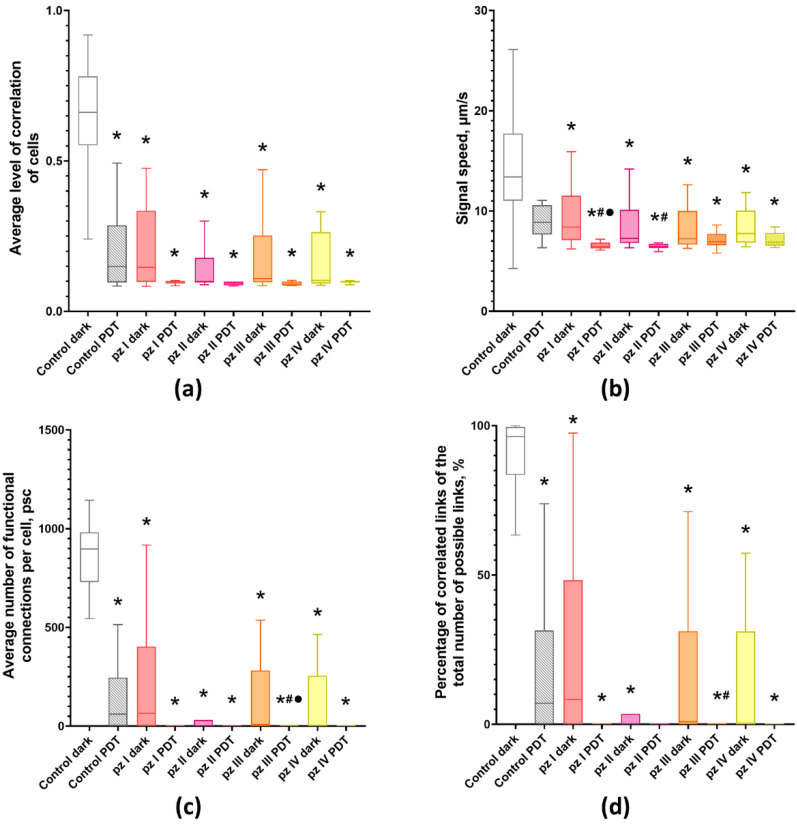
Main parameters of neuron-glial network activity in primary hippocampal cultures the next day after PDT. (**a**) Mean correlation level of cells, (**b**) signal speed, (**c**) average number of functional connections per cell, and (**d**) percentage of correlated connections from the total number of possible connections. * versus “Control dark”, # versus “Control PDT”, ● versus the same **pz** dark group, *p* < 0.05, Kruskal–Wallis test.

Therefore, the next stage of our study was focused on the analysis of the main network characteristics of primary hippocampal cultures including the average level of correlation of Ca^2+^ oscillations, the signal speed propagation, the average number of functional connections per cell, and the percentage of existing correlated connections between cells from the total number of possible connections (Figure 3).

It was shown that, normally (“Control dark” group), the cells in the neuron-glial network form multiple connections (897.18 [735.53; 976.14] connections in each cell on average) and have a high level of signal speed propagation (0.66 [0.57; 0.77] µm/s), which corresponds to the 14th–15th days of culture development in vitro [20].

Application of **pz I**–**IV** without PDT leads to significant changes in the network characteristics of neuron-glial networks. Against the background of a decrease in the number of working cells and Ca^2+^ activity profile in all experimental groups (Figure 2), there is a decrease in signal speed propagation between cells and the average level of correlation and number of functional connections per cell (Figure 3).

PDT also had a negative effect on the network characteristics of neuron-glial networks. The signal speed propagation between cells in the “Control PDT” group remains unchanged; however, there is a significant decrease in the average level of correlation (0.15 [0.10; 0.23]) and the average number of functional connections per cell (60.51 [3, 09; 165.87]). The use of **pz I**–**IV** during irradiation led to a decrease in all studied network characteristics, which indicates a pronounced disruption of the networks functioning. The most pronounced changes were observed in the **pz I**–PDT and **pz II**–PDT groups, in which a significant decrease in signal speed propagation between cells (6.45 [6.28; 6.79] and 7.27 [6.85; 9.17] µm/s) relative to the “Control PDT” group (13.39 [11.31; 17.42] µm/s) was shown. Other analyzed network parameters tended to zero (Figure 3).

**Figure 4:**
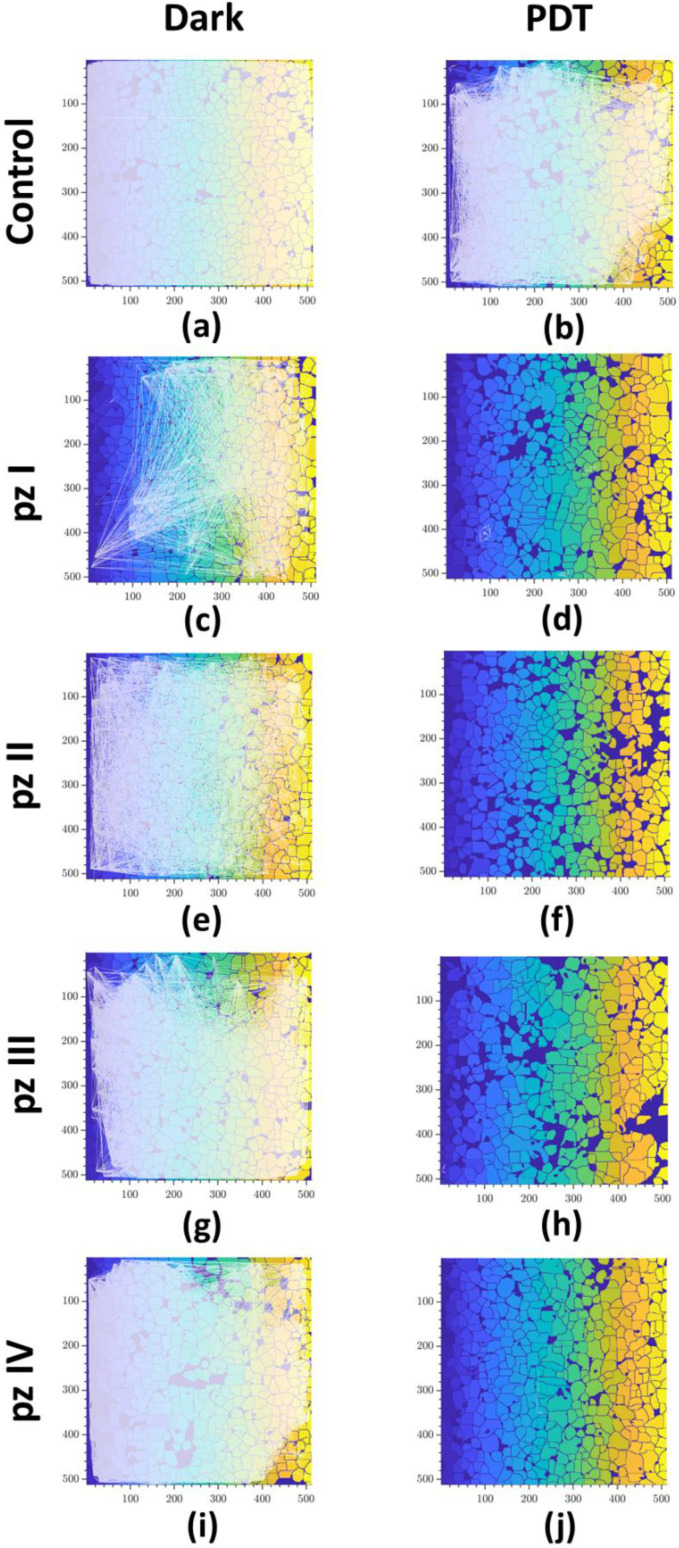
Representative correlation network graphs of primary hippocampal cultures the next day after PDT. Each white line schematically represents the connection between cells. The line is plotted on the graph if the correlation level of Ca^2+^ oscillations is greater than the empirically calculated value 0.3: (**a**) control dark, (**b**) control PDT, (**c**) **pz I** control, (**d**) **pz I** PDT, (**e**) **pz II** control, (**f**) **pz II** PDT, (**g**) **pz III** control, (**h**) **pz III** PDT, (**i**) **pz IV** control, and (**j**) **pz IV** PDT.

The construction of correlation network graphs showed that, in the normal state, the neuron-glial network of the primary hippocampal cultures has a large number of connections (Figure 4a). PDT led to the partial destruction of connections between cells (Figure 4b). A similar dynamic of changes was observed in the groups of cultures with **pz I**–**IV** application without PDT (Figure 4c,e,g,i). The most significant changes are noted in the “**pz I** dark” group. A complete destruction of the connections between the cells that form the neuron-glial network was shown 24 h after photodynamic exposure with the application of all the studied photosensitizers (**pz I**–**IV**) (Figure 4d,f,h,j).

**Figure 5:**
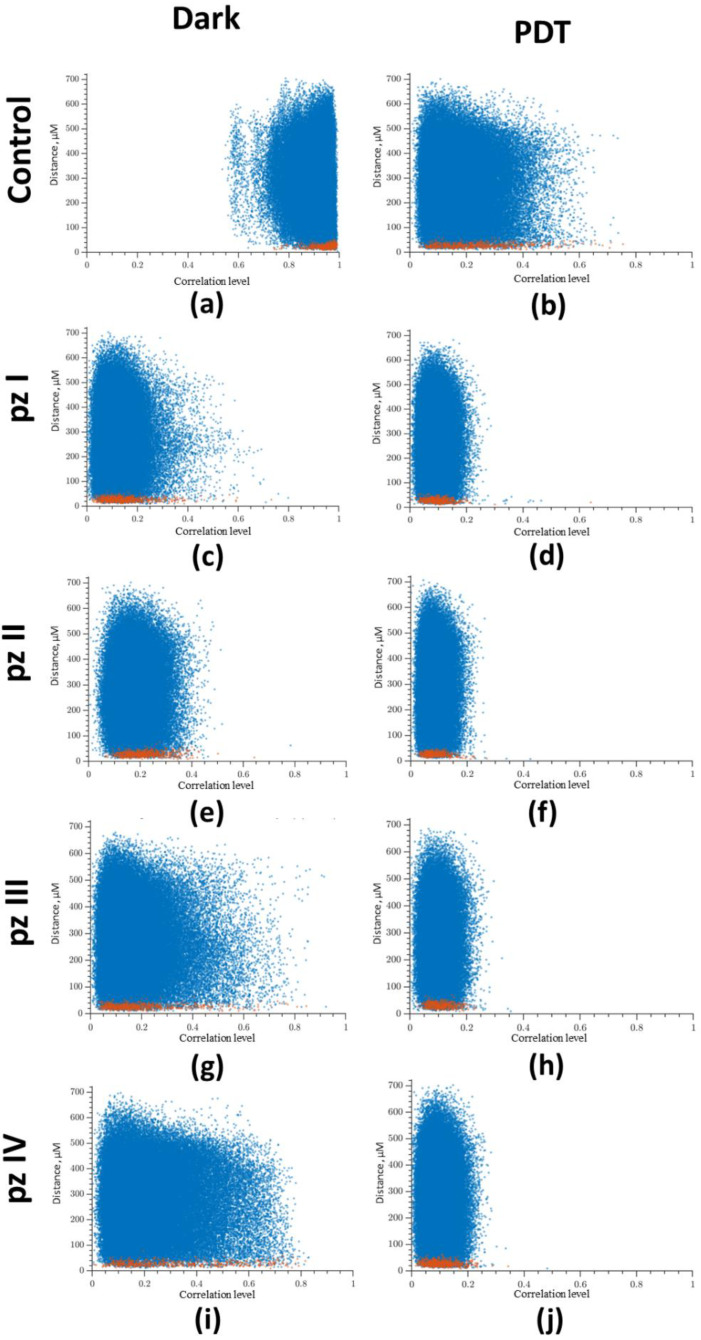
Dependence between the correlation level and the distance of cells pairs. The adjacent cell pairs with their soma in direct contact are highlighted in red: (**a**) control dark, (**b**) control PDT, (**c**) **pz I** control, (**d**) **pz I** PDT, (**e**) **pz II** control, (**f**) **pz II** PDT, (**g**) **pz III** control, (**h**) **pz III** PDT, (**i**) **pz IV** control, and (**j**) **pz IV** PDT.

Next, we analyzed the relation between the level of correlation of Ca^2+^ oscillations on the distance between cells (Figure 5), which showed a high level of correlation in the normal state (“Control dark” group). Most of the points reflecting the relationship between a pair of cells are in the correlation range from 0.8 to 1 (Figure 5a). PDT led to a significant decrease in the level of correlation between cells (Figure 5b). In the “Control PDT” group, the point cloud was shifted to the to the beginning of coordinates, and the correlation level was in the range from 0 to 0.5. A decrease in the level of correlation was also noted in the groups of cultures with application of **pz I**–**IV** without irradiation. After photoactivation of all studied photosensitizers (**pz I**–**IV**), the point cloud was concentrated in the range from 0 to 0.2, which is considered to be a lack of correlation (Figure 5d,f,h,j).

**Figure 6:**
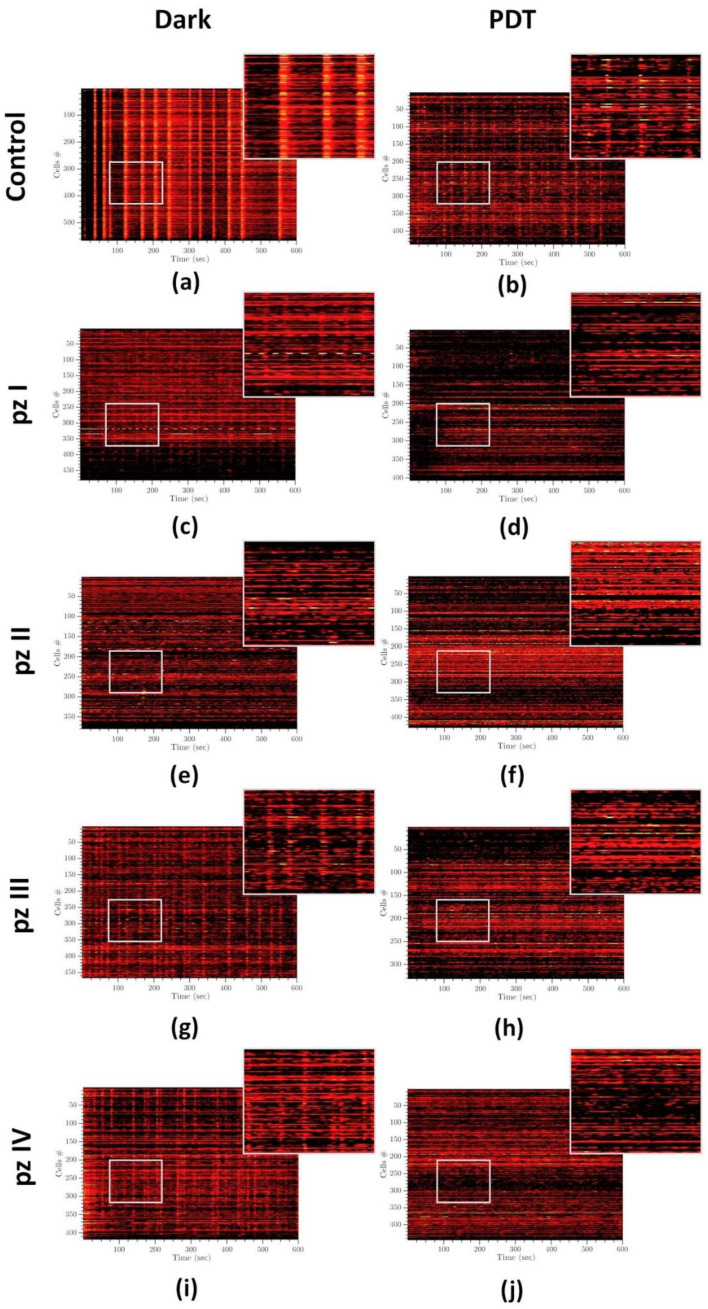
Representative raster diagrams of spontaneous calcium activity of primary hippocampal cultures the next day after PDT. The X-axis represents the time of recording; the Y-axis represents the number of active cells, respectively. The lighter the point, the more intense the fluorescence. Treatments of the following: (**a**) control dark, (**b**) control PDT, (**c**) **pz I** control, (**d**) **pz I** PDT, (**e**) **pz II** control, (**f**) **pz II** PDT, (**g**) **pz III** control, (**h**) **pz III** PDT, (**i**) **pz IV** control, and (**j**) **pz IV** PDT.

Analysis of raster diagrams of calcium activity in the “Control dark” group showed the synchronized activity of cells in the neuron-glial network. The spontaneous calcium activity profile is characterized by the appearance of superoscillations (Figure 6a). PDT significantly changed the pattern of calcium activity of primary hippocampal cultures. In the “Control PDT” group, the superoscillations were not registered; the number of grouped, synchronized Ca^2+^ events were reduced (Figure 6b). A similar dynamic of changes was observed in the groups of cultures with application of photosensitizers (**pz I**–**IV**) without irradiation (Figure 6c,e,g,i). The most pronounced changes in the profile of calcium activity were observed in the groups of cultures with the application of **pz I** (Figure 6c) and **pz II** (Figure 6e) where the profile of calcium activity was mainly represented by ungrouped single Ca^2+^ oscillations. Photodynamic exposure aggravated the effect of **pz I**–**IV** on the pattern of spontaneous calcium activity of primary hippocampal cultures (Figure 6d,f,h,j). The synchronized Ca^2+^ events were completely absent in these experimental groups.

## 4. Discussion

Despite the achievements in modern medicine, a steady increase in oncological diseases has been observed all over the world. Since the middle of the 20th century, PDT has been a clinical option for the treatment of tumors of various origins [6,7,14,32]. In particular, along with chemotherapy and fractionated radiotherapy, PDT is regarded as a therapeutic modality for the treatment of gliomas as well as for fluorescent-guided brain tumor resection [33,34,35]. Moreover, in the context of the modern paradigm on the critical role of the immune system in cancer treatment, accompanied by the active development of personalized immunotherapeutic anti-cancer strategies, the attractiveness of the PDT approach is also associated with an ability to induce regulated cancer cell death modalities with immunogenic properties, which provides endogenous stimulation of antitumor T-cells and the generation of long-term immunological memory, and eventually allows us to achieve complete tumor eradication and negates the risk of metastasis [7,17]. However, the controversial evidence of PDT efficiency on overall survival of patients with gliomas as well as persistent side effects, technical limitations in light delivery, and photosensitizer design do not allow for the widespread use of this approach in the clinic as a standard treatment mode [14]. Nevertheless, in a view of the promising prospects of PDT in anti-cancer therapy, the intensive research aimed at boosting PDT efficiency continues [36]. Special attention is given to the design of novel photosensitizers with improved photodynamic properties and a low systemic toxicity effect for normal tissues.

Representatives from tetrapyrrole macrocycles whose photodynamic properties are widely discussed in the literature are of particular interest [37,38,39,40]. These compounds have improved the absorption and retention properties as well as “open” pyrol sites for substitution [41]. Substitution with aryl- and cyano- groups enables the design of tetracyanotetra(aryl)porphyrazines, which support the high photodynamic activity and have unique properties of molecular rotors [42]. Previously studies have shown that PDT leads to a significant increase in intracellular viscosity of tumor cells [43,44]. The fluorescence of molecular rotors depends on the rate of intramolecular rotation. In high viscosity media, intramolecular rotation is inhibited resulting in a significant increase of the emission intensity and the fluorescence lifetime. The assessment of viscosity characteristics by measuring the lifetime of fluorescent molecular rotor excitation using time-resolved microscopy allows for early monitoring of tumor cells’ sensitivity to photoinduction, and the efficiency of cell death during PDT procedure or immediately after its termination. The functions of molecular rotors were shown for **pz I**–**IV** in our previous studies [18,27,28,42,45,46]. That means that **pz** can provide promising prospects for personalized PDT dosimetry aiming to achieve the highest treatment efficacy with minimal cytotoxic effect for surrounding normal tissues. Moreover, the attractiveness to **pz** for PDT glioma treatment also lies in their ability to induce immunogenic cell death that was previously shown in vitro and in vivo in a tumor prophylactic vaccination model [47]. This indicates a high potential of the use of **pz** not only as a personalized PDT dosimetry tool, but also as an effective PDT treatment mode.

We have previously shown that **pz I**–**IV** effectively kills murine glioma GL261 cells and causes different long-term dark toxicity effects on primary neuronal cultures [18,19]. Herein, we assessed the influence of **pz I**–**IV** on the functional network activity of primary hippocampal cultures in a normal state and after PDT. Carried out experiments revealed that PDT without the application of **pz** leads to a significant decrease in the main parameters of the functional calcium activity of neuron-glial networks and causes a negative effect on the network characteristics, resulting in the partial destruction of connections between cells, a decrease in the level of correlation between cells, and simplification of the pattern of calcium activity. Similar dynamics of changes in calcium activity parameters and network characteristics were observed in the application of **pz I**–**IV**. PDT aggravated the negative effect of **pz I**–**IV**. Against the background of a dramatic decrease in the number of metabolically active cells accompanied by a decrease in the frequency and an increase in the duration of Ca^2+^ oscillations, there was a decrease in all studied network characteristics and pronounced simplification of the spontaneous calcium activity profile 24 h after irradiation. The observed effects suggest that, even in the absence of pronounced toxic effects on morphological integrity, the accumulation of **pz I**–**IV** in healthy nerve cells can cause significant changes in the functional architectonics of neuron-glial networks, leading to impairments in synaptic transmission and degradation of axons and dendrites, which in turn can potentially result in the complete destruction of neural networks and, consequently, loss of brain functions. We previously found that **pz I**–**IV** were substantially accumulated in neurons and glial cells in primary neuronal cultures mostly in lysosomes (**pz I**–**IV**) and partially in the endoplasmic reticulum (ER) (for **pz I** and **pz II**) even two hours after **pz** addition to the culture medium [19]. It could be assumed that accumulation of **pz** in the lysosomes can trigger a lysosomal stress response accompanied by the release of Ca^2+^ ions through calcium permeable channels into the cytosol [48]. Moreover, the lysosome resident protein the mammalian target of rapamycin (mTOR) contributes to the stress of the ER, the main depot of Ca^2+^ in cells [48]. Thus, both lysosomal and ER stress can initiate the development of excitotoxic effects, which significantly affect the spontaneous calcium activity of nerve cells. The formation of reactive oxygen species under PDT apparently accelerates and intensifies the effects of lysosomal and ER stress leading to dramatic consequences in the spontaneous calcium activity of primary hippocampal cultures.

Therefore, considering the significant changes in the functional activity of neuron-glial networks in vitro, the feasibility of the direct application of PDT based on **pz I**–**IV** in the therapy of gliomas is highly controversial. Nevertheless, the unique properties of **pz I**–**IV** retain a great prospect of their use in PDT cancer therapy. Looking ahead, **pz I**–**IV** could be applied in the therapy of other types of tumors differed by cells’ origin and cellular metabolism from glioma and healthy nervous system cells. For instance, we previously showed that **pz II** and **pz IV** cause pronounced cytotoxicity against human epidermoid carcinoma A431 cells, whereas photoinduced effects regarding human immortalized keratinocytes HaCaT were significantly lower [18]. PDT based on **pz I** and **pz III** effectively kills fibrosarcoma MCA205 cells through the immunogenic pathway accompanied by the emission from PDT-induced cancer cells of two crucial DAMPs (ATP and HMGB1) with subsequent engulfment by antigen-presenting cells (i.e., dendritic cells, DC) and induction of their activation and maturation in vitro [47]. Moreover, in the tumor prophylactic vaccination model *in vivo*, it has been shown that fibrosarcoma MCA205 cells stimulated with **pz I**-PDT or **pz III**-PDT served as a potent vaccine-activated adaptive immune response and significantly decreased the tumor growth at the challenge site [47].

Currently, the development of DC-based vaccines is regarded as an alternative approach for the treatment of gliomas that allows for avoiding pronounced toxic effects for a healthy nervous system [49]. The most common design of DC vaccines is the application of specific antigen peptides/RNA for pulsing DCs or whole-glioma tumor cells killed via freeze/thawing-based necrosis [50]. However, although the former methodology might exhibit low efficacy due to the high antigenic heterogeneity of brain tumors, the latter procedure is associated with poor immunogenic potential. Considering the evidence that **pz**-based PDT can trigger ICD [47], it could be assumed that application of PDT-induced glioma cells for pulsing DCs will significantly improve the immunogenic potential and the ability to induce a superior T helper 1-mediated immunity, that in turn allows for achieving a high efficacy of a new generation of DC vaccines against gliomas. Indeed, this is an intriguing direction for future research, and more detailed experiments in vitro and in vivo are expected.

## 5. Conclusions

We characterized the effects of four photoactive dyes of the tetracyanotetra(aryl)porphyrazine group (**pz I**–**IV**) on the functional activity of neuron-glial networks in primary hippocampal cultures in their application in normal conditions and under PDT. It was shown that application of **pz I**–**IV** leads to a significant decrease in the main parameters of the functional calcium activity of neuron-glial networks and causes the pronounced changes in the network characteristics. The observed negative effects of **pz I**–**IV** were aggravated under PDT. Considering the significant restructuring of the functional architectonics of neuron-glial networks that can lead to the severe impairments in synaptic transmission and loss of brain functions, the feasibility of direct application of PDT based on **pz I**–**IV** in the therapy of brain tumors is highly controversial. Nevertheless, the unique properties of **pz I**–**IV** retain a great prospect of their use in the therapy of tumors of another origin and cellular metabolism.

## Data Availability

The data used to support the findings of this study are available from the corresponding author upon request.

## Abbreviations

DAMPs	damage-associated molecular patterns
DC	dendritic cell
DIV	day of culture development in vitro
ER	endoplasmic reticulum
ICD	immunogenic cell death
ROS	reactive oxygen species
PBS	phosphate-buffered saline
PDT	photodynamic therapy
**pz**	tetracyanotetra(aryl)porphyrazines
BBB	blood–brain barrier
